# Patient Care During the COVID-19 Pandemic: Use of Virtual Care

**DOI:** 10.2196/20621

**Published:** 2021-01-21

**Authors:** Andy Wong, Rashaad Bhyat, Siddhartha Srivastava, Lysa Boissé Lomax, Ramana Appireddy

**Affiliations:** 1 Division of Neurology Queen's University Kingston, ON Canada; 2 Canada Health Infoway Toronto, ON Canada; 3 Department of Medicine Queen's University Kingston, ON Canada

**Keywords:** virtual care, teleneurology, telemedicine, medical informatics, internet, patient-physician relationship, email, digital health

## Abstract

Virtual care, the use of videoconferencing technology to connect with patients, has become critical in providing continuing care for patients during the current COVID-19 pandemic. Virtual care has now been adopted by health care providers across the spectrum, including physicians, residents, nurse practitioners, nurses, and allied health care professionals. Virtual care is novel and nuanced compared to in-person care. Most of the health care providers who are delivering or expected to deliver virtual care have little to no prior experience with it. The nuances of virtual care involve regulatory standards, platforms, technology and troubleshooting, patient selection, etiquette, and workflow, all of which comprise critical points in the provision of health care. It is important to consistently deliver high-quality, equitable, and professional virtual care to inspire patients with the trust they need to continue follow-up of their care in these difficult times. We have been adopting virtual care in our clinical practice for over two years. In partnership with Canada Health Infoway, we have assembled a primer for virtual care that can serve as a guide for any health care provider in Canada and globally, with the goal of providing seamless transitions between in-person and virtual care.

## Introduction to Virtual Care

As part of the response to the COVID-19 pandemic, health care organizations across Canada have cancelled elective and nonurgent clinics as a measure to reduce the risk of exposing patients to COVID-19 [1]. To provide safe, timely, and accessible ambulatory care, health care providers have adopted virtual care in Canada and globally [2-7]. Virtual care can be defined as any interaction between patients and members of their circle of care that occurs remotely, using any form of communication or information technology, to facilitate or maximize the quality and effectiveness of patient care [8-11].

## Role of Virtual Care in Addressing Barriers to Health Care

Virtual care is novel and nuanced compared to in-person care. Virtual Care has significant benefits to patients, health care systems and society at large by offering patient-centered care [12-16]. Virtual care also helps increase health care capacity by optimal use of health care professionals’ time, infrastructure, and reduced per capita health care cost [13]. Virtual care is more relevant during the COVID-19 pandemic because it provides access to medical care that is timely, convenient, efficient, and safe with reduced risk of transmission [17]. Despite all these advantages of virtual care, it is important to ensure that virtual care continues to provide equitable access to health care. Virtual care has the potential to remove some of the current barriers to health care with regard to availability, accessibility and affordability [13,18-20]. Geographical distance, travel burden and out-of-pocket expenses are some of the largest barriers to ambulatory health care access, and virtual care has great potential to address these [2,21]. However, sociodemographic characteristics such as age, sex, gender, level of education, and English proficiency, as well as socioeconomic status indicators such as race and ethnicity, can impact one’s ability to access and use the technology needed for participating in virtual care [22-24]. Virtual care has the potential to provide access to people who live in more rural areas, who tend to have lower socioeconomic status, are older, and have lower levels of education, but only if the patient can access video and audio equipment, spend the time to use and troubleshoot the equipment, and learn to use programs and hardware they may have never used before. There is a need for engagement and commitment from all stakeholders in the health care system to ensure that virtual care can successfully provide equitable access, closing the gap between rural and urban health care [25].

The Division of Neurology at Queen's University/Kingston Health Sciences Center has been an early adopter of virtual care for ambulatory services since 2016 for stroke, epilepsy, and sleep medicine, and it has an active virtual care research program [13,26]. Leveraging our long experience with virtual care, the Southeastern Ontario Academic Medical Organization and Kingston Health Sciences Center have enabled a rapid rollout of virtual care services for its 450 physicians, 575 medical residents, and 160 allied health care professionals over two weeks using the Reacts platform [27]. Many health care providers (physicians, residents, medical students, nurses, and allied health care professionals) are not familiar with using virtual care and have identified virtual care and digital health technologies as areas of emerging educational and training focus [9-11,27,28]. Here, we present a comprehensive, practical guide for any health care provider wishing to provide virtual care.

## Guide to Virtual Care

### Licensing and Regulatory Standards

At this time, no rigorous standalone virtual care guidelines have been established by licensing bodies in Canada [9]. Some guidelines have been temporarily developed as a response to the COVID-19 crisis and refer to pre-existing telemedicine guidelines that may require rejuvenation [29,30]. The Canadian Medical Association is providing guidance through the Virtual Care Playbook [31]. Provincial and territorial professional organizations for physicians have released some local guidance on virtual care [3,32-35]. In addition, an excellent pan-Canadian list of virtual care resources is available through the Royal College of Physicians and Surgeons [36].

Further information on medicolegal liability can be obtained from the Canadian Medical Protective Association (CMPA) [37,38]. Nonphysician health care providers (nurses, nurse practitioners, and other allied health care staff) are advised to refer to their own professional and licensing bodies for further guidance. It may be reasonable to refer to the above resources for general guidance on providing virtual care.

### Types of Virtual Care and Platforms to Use

Secure messaging, secure email, and secure video conferencing are some of the most used virtual care modalities. These services can be delivered on multiple platforms and devices. The platforms are broadly divided into regulated and unregulated categories. Regulated platforms are those that comply with Canada's federal Personal Information Protection and Electronics Document Act (PIPEDA), as well as provincial and territorial privacy laws that apply to health or medical records, such as Ontario's Personal Health Information Protection Act (PHIPA). A comprehensive list of regulated platforms in Canada is available [3,36]. Unregulated platforms include those that do not meet the federal and provincial/territorial privacy standards (FaceTime, WhatsApp, Google Duo, etc).

For the virtual care provider, the choice of platform may depend on the features offered, such as secure messaging, video conferencing, patient portals, integration into electronic health records (EHRs), and integration with other remote monitoring devices and applications. Before finalizing a decision on a platform, we recommend that practitioners seek clearance from their own organization's privacy and security officer if available. Each organization and each individual provider will have a unique set of circumstances, resources, and abilities. It is essential for each provider to develop familiarity with the platform selected by their organization. The platforms currently used at Queen's University are the Ontario Telemedicine Network and Reacts [27].

### Digital Privacy

It is important to ensure that the appropriate precautions are taken by the physician and the patient to ensure the privacy of health care information [39]. For additional information on privacy, security, and data stewardship considerations relating to digital health technologies in the outpatient setting, clinicians should consider guidance from the College of Family Physicians of Canada and the CMPA or from local or regional professional associations [40,41].

### Equipment

The equipment (or hardware) required for delivering virtual care is widely available and includes smartphones, tablets, laptop computers, and desktop computers. Ideally, all these devices should have built-in or peripheral hardware for video conferencing (web camera, speaker, and microphone). The network (bandwidth) requirements must be confirmed before use, although most of these platforms enable the quality of the video conference to be adjusted to low bandwidth. In general, the faster and more reliable the internet connection, the better the experience will be for the provider and the patient.

The choice of the device used by the provider depends on multiple factors, including the physical location of the provider at the time of the virtual video visit. Although virtual visits can be conducted from any internet-enabled device, we suggest using a laptop or a desktop computer with a built-in camera and speaker. These devices typically provide the highest quality internet connection, a large screen, and a better speaker and microphone setup. However, this is highly dependent on the technology available to the patient, as modern-day tablets and smartphones have high-quality built-in cameras and speakers. Perhaps most importantly, the patient and provider should be familiar with their respective devices in case troubleshooting is required.

### Patient Selection for Virtual Care

It is essential to identify when virtual care is most appropriate for patients. Virtual care is an effective alternative to in-person care for many but not all medical conditions and clinical scenarios. Some general guidelines on patient selection and screening are provided in table 1 [13]. Given the nuances of clinical medicine, with its multiple specialties and subspecialties, health care providers should use professional judgement when deciding which patients, medical conditions, and types of clinical encounters are most suitable for virtual visits [29,38]. As a general guide, virtual care is ideal for nonacute or routine clinical activities, such as reviewing test results and symptom follow-up.

**Table 1:** Guide to virtual care channel selection.

Virtual care channel	Ideal patient	Ideal conditions	Nonideal conditions
Video	Has access to required technology and the internet Is comfortable with using video technology Has no physical/sensory/cognitive disabilities that would limit videoconference use (unless supported by family and/or friends)	All clinical conditions or encounters that do not need hands-on physical examination Follow-up of test results Counselling Discussing treatment options Discussing prognosis Follow-up of symptom severity	Workup of complex physical conditions needing physical hands-on examination for diagnosis and therapeutic planning
Secure messaging/email/chat	Has access to email/chat Is comfortable with using the modality Has physical or sensory disabilities that make video conferencing/in-person/telephone difficult Has a busy work schedule	Follow-up of routine test results that are normal and do not require intervention Answering patient questions about medications Conditions that need very frequent ongoing follow-up Communication regarding upcoming test results	Initial encounters with patients Workup of new symptoms, especially complex ones Discussing complex diagnostic/therapeutic plans Initiating new treatments that are complex Breaking bad news Providing prognostic information on major medical illness
Telephone	Has barriers to technology/internet access Lacks comfort using video technology	Follow-up of test results Follow-up of symptom severity Follow-up of treatment response Counselling Discussing treatment options Treatment titration Discussion of prognosis	Initial encounters with patients Workup of new symptoms, especially complex ones

### Virtual Care Workflow: Registration, Appointment, Scheduling, and Documentation

It is mandatory in all jurisdictions to document all health care encounters as part of the patient's health care record. Provincial and territorial medical regulatory authorities and the CMPA consider appropriate documentation to be an essential communication skill and a core component of physicians' professional best practice.

Although some virtual care platforms integrate the video visit into the provider's EHR, patient portal, or hospital information system, this practice is not yet commonplace. Thus, two parallel workflows often coexist for the provider: the first for the virtual care platform, and the second for the provider's medical record system.

This “double workflow” ensures that the virtual care encounter details (registration, clinical documentation, follow-up plans, planned investigations, and therapeutic plans) are captured in the patient's health record.

### Consent

It is necessary practice to discuss consent with the patient at the time they sign up for virtual care, covering subjects such as the inherent risks related to privacy and security as well as the limitations of virtual care [32]. There are two routes by which patient information could leave the circle of care. One route is the “real world.” Nonprivate, nonstandard locations on both the patient’s and clinician’s side are now used; this increases the risk that another party will hear the discussion, whether accidentally or intentionally. The precautions described within this article will help to reduce this risk. The other route is the “virtual world,” where there is an inherent risk in the web-based connection between the two parties. Internet and virtual care security have come a long way from their beginnings, and the weak link in information security often lies in the user’s hands. All our connections from the clinician’s side necessitate standard password complexity and 2-factor authentication, including on personal devices that may be used remotely. We also only use software designed and approved for the exchange of personal health information.

The limitations of virtual care should be made clear to the patient. It should be clear that when the clinician cannot see the patient physically, there is inherent risk of missing physical findings. This is only exacerbated by the potential for unstable network connections or poor quality/malfunctioning equipment. It should also be made clear to patients that with this in mind, should there be a requirement for in-person assessment, there may a delay relative to the traditional method of seeing the patient in-person. The option of seeing a clinician in person should always be given to the patient and should always be suggested by the clinician if they feel that the virtual visit was insufficient for the patient’s care or may even have caused harm.

Generic consent forms are readily available on the web through many of the resources referenced in this paper [31,37,39], although consent is often obtained verbally given the context and must be documented in the subsequent notes. Although regulated health care software applications require the patient to consent at the time of account signup, informed consent should be obtained from the patient or substitute decision maker before beginning any virtual care interaction.

### Patient Setup and Education

It is crucial to gauge the degree of a patient's or caregiver's comfort level with using technology before proposing the use of virtual care. Some health conditions can affect motor strength, coordination, language, vision, and cognition. All these challenges can adversely affect a patient’s ability to use personal internet-enabled devices and participate in virtual care, even if the patient is proficient with technology. In addition, it is important to recognize that not all patients will have easy access to the ideal technology for virtual care, including video capability and a sufficiently fast internet connection. We have found that modern mobile phones are capable and ubiquitous but may have poor video and/or audio quality depending on the internet connection. Some patients have chosen to use audio-only telephone visits instead. As we transition to virtual care, we need to be keenly aware of patient-readiness for this transition. Although audio-only visits are shown to be useful, an unexpected change to an audio-based visit from a planned video-based visit may result in slight inconvenience at best and a delay in essential visual assessment and therefore care at worst. In cases of communication breakdown, an in-person visit is often suggested.

With the patient's consent, providers should explore the option of having a patient's family member or friend assist with facilitating a virtual visit [13]. Patients and their families who are interested in virtual care should be provided with information and education about signing up for and using the service.

It is also helpful to provide some tips for the patient to prepare for the virtual care visit. The patient’s clothing should allow a reasonable range of motion if the provider requests physical maneuvers during the virtual visit. If the patient requires a hearing or visual aid, these should be readily available. For patients with hearing, visual, or cognitive challenges, it is often more challenging to see and hear via a screen and speakers, in contrast to an in-person visit. Patients requiring mobility aids should keep these devices close by and may benefit from the presence of a nondisabled individual to assist them as necessary.

### Etiquette

Many health care providers are not familiar with how their patients will perceive them in a virtual visit. With this in mind, it is useful to be aware of the etiquette that is unique to virtual care [42,43]. The basic principles of developing a high-quality “webside manner” can be separated into two areas: technical considerations and communication skills.

#### Technical Considerations

Due to technical limitations, auditory and visual information can be lost during a virtual consultation. At the beginning of the session, both cameras should be directed so that the faces and shoulders of both the patient and the health care provider are framed. Remember that slower, broader movements decrease visual blurring, and obscuring the face and mouth should be avoided. The physician should be mindful that gazing at the patient's image on the screen can make it seem as though the physician is looking down at them. When possible, the physician should look into the camera.

#### Communication Skills

During the session, the interviewer must recognize the importance of clear communication. Virtual visits require communication adjustments. Physicians should speak slowly and use nonmedical terms for maximum clarity. They should be prepared for lags in communication for technical reasons. Recognizing these technical limitations reinforces the need for speaking clearly. It may be helpful for the interviewer to check in with the patient to see if they can hear and understand their messaging. Reiterating and summarizing what a patient has said may also be helpful. In addition, clear signposting by the interviewer can indicate the progress and direction of the session. For example, the interviewer could say, “I am now going to summarize what I understand of your history, then proceed to the physical exam.”

The primary sensory modalities available in a virtual visit are limited to auditory and visual. However, touch is a standard method of communication during an in-person visit, especially during the physical examination. When requesting that patients perform physical examination maneuvers during a virtual visit, adjustments are required. The clinician should clearly state the movement that the patient is asked to perform. They should consider documenting the absent physical examination maneuvers that would have typically been included but were not feasible due to the virtual nature of the consultation. This suggests, at a minimum, that these steps have been considered. Also, the clinician should inform the patient before they navigate to another screen to look at laboratory or imaging data. When possible, screen-sharing modes should be used to show patients their images or laboratory results.

Last but certainly not least, kindness and compassion are paramount in a virtual visit. Listening carefully to a patient's concerns, taking time to clarify their statements, and following up on their questions are vital skills, regardless of what form of medical care is being offered.

### Environment and Setting

Preparing one's virtual visit environment is vital for both patients and providers. Clinical administrative staff play a key role. Prior to the appointment, the staff should send a message to the patient with recommendations on setting up for a visit. Some recommendations are relevant to patients and virtual visit providers alike:

- The room should be reasonably private, with minimal potential for distractions. It may be helpful to notify other members of the household or building that space will be used privately for the duration of the appointment. If possible, adequate lighting, presenting oneself in an uncluttered room, and ensuring the visibility of one's face and shoulders are ideal. Background should be reduced to a minimum. This may include distancing from a louder area (kitchen, public area, open windows, etc). We recommend posting a notice at the entrance to the room indicating that a confidential meeting is occurring. Personal communication devices (ie, mobile phones and pagers) should be switched to silent mode to avoid distraction.
- If other parties are involved in the meeting, such as trainees, family members, and friends, they should be introduced prior the beginning of the meeting. As with standard in-office visits, the people who are involved in the meeting should be transparent and within the control of the patient and physician.

Other recommendations are more relevant to patients and can be tailored to their medical conditions and clinical scenarios (eg, postoperative vs chronic disease follow-up):

- For some clinical specialties, it may be helpful for the patient's room to have enough space to allow them to stand and move away from the camera, so their entire body is visible.
- It is helpful if the camera is high-definition (720p or higher) and wide-angle. The room should have a comfortable yet easily movable chair available for the patient to rest.
- If the consultation requires equipment, the patient should have an easily accessible area to place these materials.
- A high-quality internet connection within the selected room is also essential. A wired connection is ideal, but if only Wi-Fi is available, the room should not be too far from the wireless router to ensure a reliable connection.

### Example Run-through of a Virtual Visit

#### Presession Preparation

During the presession preparation, attention must be paid to the equipment, environment, and external appearance. In addition to checking the equipment and network, it should be ensured that the general guidelines laid out in the above section with regard to etiquette and the environment are followed. The patient's information or EHR should be available within reach of where the video visit is happening.

A good habit is to check the platform appointment software to confirm that the patient or patients have been booked as expected. If there is an issue with connecting to the patient, it is always useful to have an alternative means of communication. Ideally, this will be a telephone number; however, email or instant messaging can be used. Most platforms allow the patient to join and wait in a virtual “room.” It is recommended to mute the microphone before the patient enters the appointment.

#### Session

The first thing to do when the patient logs in is to confirm that both parties’ cameras and microphones are working correctly and troubleshoot as necessary. In all first visits, it should be expected that there will be delays due to technological issues. Technical factors play a significant role in one’s ability to communicate effectively with a patient. It is vital for the medical professional to be familiar with the platform they are using, as it is not uncommon to have to walk through teaching a patient how to modify camera or audio settings. It is essential to explain the steps in a simple and organized fashion.

Formal introductions can be made next, in which it is essential to obtain consent for the virtual visit and establish the identity of the patient. The ID verification step may not be necessary for clinical scenarios in which the patients are well known to the interviewer (eg, family practices, specialist practices managing chronic diseases). Announcing who is involved in the meeting for both parties is useful, as some participants can be virtual and others can be physical but not visible (off-camera).

The history will likely feel very similar to an in-person visit. It is useful to be clear and intentional with questions, as information can easily be lost through the virtual connection. The physical examination will be a different experience, as traditional medical training in conducting examinations anticipates that the patient will be physically present. When the correct group of patient, diagnosis, and visit purpose are selected, a virtual physical examination is equivalent to an in-person visit examination. Investigations can be reviewed with the patient, including sharing the actual results and images directly via screen sharing. If discussions with colleagues or other staff are necessary, the patient may be notified and muted. At the end of the appointment, as with most appointments, the clinician should discuss and establish a plan. Most relevant in this context is whether a follow-up appointment will be a virtual visit or in-person visit. Because the patient will not be taking any items (prescriptions, requisitions, instructions, etc) with them, it is crucial to verify details with the patient (eg, pharmacy, next steps).

#### Postsession

If orders and prescriptions are integrated into the EHR, clinicians can follow their routine workflow. Otherwise, orders and prescriptions must be sent post-encounter (eg, secure messaging, email, electronic prescriptions such as PrescribeIT, faxes, or mail). It should be ensured that follow-up arrangements have been made. Finally, the encounter can be documented by clinicians as per typical workflow (handwritten, dictation, or typed in the EHR), clearly identifying the visit as an eVisit or virtual visit.

Textbox 1 demonstrates a checklist that summarizes what we have discussed in the above sample run-through visit. It forms a guideline to maintain the quality of a virtual visit across all patients but also serves as a tool to structure future quality improvement projects.

**Checklist for virtual care.**

Before the virtual video visit (presession):

- Check that the device, video conferencing equipment, and network are working properly
- Ensure that the camera and microphone are positioned properly
- Ensure that the room is well lit and does not contain any distractions and that only professional objects are visible
- Ensure privacy during a virtual video visit:
  Mute your mobile phone or place it on vibrate
  Close the office door and place a “Do Not Disturb” or “Doing Video Visits” sign
- Ensure beforehand that you have access to the patient charts, results, and any other health information
- Mute the microphone until the videoconference starts
- Ensure that the patient’s telephone number is available should it be necessary

During the virtual video visit:

- Confirm that both parties’ videoconferencing technology is working and optimized for sound and video
- Introduce yourself and bring your photo ID close to the camera for the patient to see
- Ask the patient to show a valid photo ID (driver’s license or Health Card)
- Obtain verbal consent for the virtual visit
- Ask the patient to ensure their room is well lit and has enough space for movement
- Ask the patient to ensure privacy during the virtual visit:
  Mobile phone(s) are placed on mute or vibrate
  Doors are closed and indicate “Do Not Disturb”
- Ask the patient to adjust their position so they are framed adequately within the camera view
- Ensure that the patient has all objects and tools necessary for the full assessment
- Introduce any other health care providers or learners present in the room

After finishing the virtual video visit:

- Ensure that all reports and clinical documentation are complete
- Ensure that the planned investigations and medication prescriptions are ordered
- Ensure that follow-up plans have been made

## Conclusion

Virtual care will become a reality in many future medical practices, and the COVID-19 pandemic is serving as a catalyst for this impending change. Virtual care is effective at removing barriers to access, especially to specialists who are typically located in larger centers, for the remarkably distributed rural and remote patients across Canada. As we have outlined, there are important nuances in the application of virtual care that help ensure patients receive at least equivalent care, or in some ways, more effective care than that received in the traditional in-person format. Many clinical organizations are working toward establishing baseline principles, guidelines, and protocols to guide Canadian physicians. This paper is intended to contribute to an emerging Canadian body of knowledge and to support optimal professional practice in an emerging and rapidly evolving field. Most importantly, virtual care should not fragment care. It should support both high-quality care, the foundation of which is an established trust-based physician-patient relationship, and continuity of care.

## Abbreviations

CMPA: Canadian Medical Protective Association
EHR: electronic health record
PIPEDA: Personal Information Protection and Electronics Document Act
PHIPA: Personal Health Information Protection Act
